# Recent advances in computational antimicrobial peptide discovery through big data, modeling, and artificial intelligence and their interplay in ushering the next golden era of drug development

**DOI:** 10.3389/fbinf.2026.1749404

**Published:** 2026-03-17

**Authors:** Tope Abraham Ibisanmi, Xiaotao Jiang, Mark Willcox, Naresh Kumar

**Affiliations:** 1 School of Chemistry, University of New South Wales, Sydney, NSW, Australia; 2 School of Clinical Medicine, UNSW Medicine & Health, University of New South Wales, Sydney, NSW, Australia; 3 School Optometry and Vision Science, University of New South Wales, Sydney, NSW, Australia

**Keywords:** antibiotic resistance, antimicrobial peptides, bioinformatics, deep learning, drug discovery, large language models, machine learning, molecular docking

## Abstract

The accelerating antimicrobial resistance (AMR) crisis continues to render more and more conventional antibiotics ineffective. Antimicrobial peptides (AMPs) are promising alternatives to traditional antibiotics due to their broad-spectrum activity, diverse mechanisms of action, and lower propensity for resistance. Traditional discovery approaches face limitations arising from the vast sequence space and the challenge of balancing efficacy with low toxicity. Addressing these challenges is critical for developing next-generation antimicrobial agents, and computational methods are increasingly driving progress. Public repositories, and techniques such as molecular docking enable *in silico* evaluation of peptide target interactions, identifying candidates with strong binding potential. Molecular dynamics (MD) simulations offer deeper insights into how AMPs disrupt membranes, form pores, or act synergistically, while Steered MD extends this to probing membrane penetration. Artificial intelligence (AI) methods, including machine learning and deep learning, capture complex sequence activity relationships, predict novel AMPs from genomic and metagenomic data, and design new peptides *de novo* using generative models. Despite rapid advances, most existing reviews treat these approaches in isolation, leaving a fragmented understanding of their interplay. This paper addresses that gap by unifying computational strategies, highlighting synergies, and critiquing limitations. Ultimately, integrating these methodologies offers a path toward more efficient AMP discovery to fight AMR.

## Introduction

Antimicrobial resistance (AMR) is outpacing our ability to develop effective therapies. The accelerating AMR crisis continues to render more and more of the conventional antibiotics ineffective. AGAR, the Australian Group on Antimicrobial Resistance, recently published a survey documenting continued resistance to important antimicrobial agents among many of the sampled isolates (Gottlieb et al., 2018). In the United States, more than two million people are infected every year with antibiotic resistant bacteria, and about 23,000 of these people die because of these infections (CDC, 2025). A projection on the global scale indicates that by 2050 (Naghavi et al., 2024), this number might reach 10 million.

**FIGURE 1:**
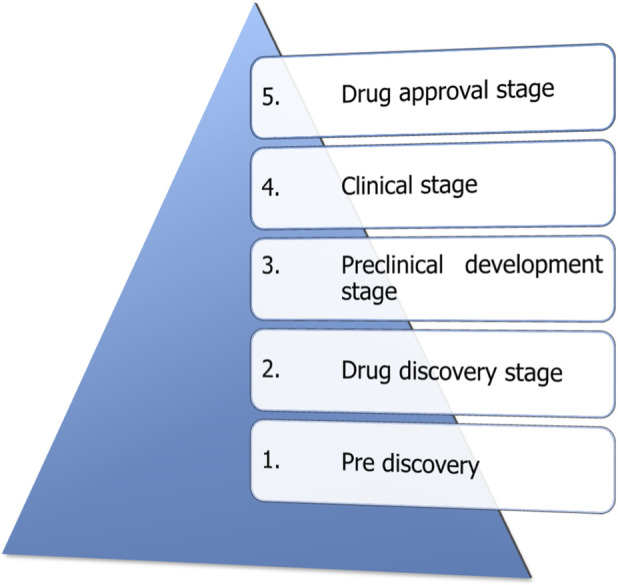
Different stages of drug development. Created in BioRender. Allwell, E. (2025) https://BioRender.com/pc1hk3o.

This highlights the need for the discovery of new and effective antibiotics, alternative therapeutics, and strategic approaches to combat drug resistance. This urgency reflects a broader challenge in drug development. Despite decades of progress, a drug discovery process, undergoes five stages (Figure 1), and estimates indicate that the probability of approval for candidate drugs entering clinical trials has steadily declined in recent years. Consequently, the research and development cost per approved drug has risen sharply.

Antimicrobial peptides (AMPs) are of great interest as alternatives to traditional antibiotics given their broad spectrum activity, mechanism of action, and lower tendency to induce resistance (Naghavi et al., 2024; Yasir et al., 2018). AMP mechanisms of action often involve interactions with microbial membranes. Most characterized AMPs form amphipathic α helices with a hydrophobic face flanked by basic residue (Oliveira Júnior et al., 2025). This amphipathic structure allows them to bind strongly to negatively charged bacterial membranes. Several mechanistic models have been suggested to explain how antimicrobial peptides disrupt membranes. In the barrel-stave model, peptides insert perpendicularly into the lipid bilayer and assemble into transmembrane pores. In contrast, the toroidal pore and carpet models describe peptides aligning parallel to the membrane, coating or bending the bilayer surface (Oliveira Júnior et al., 2025). The structural motifs of these peptides are believed to promote insertion into bacterial membranes, which are enriched in anionic lipids, thereby compromising membrane integrity (Naghavi et al., 2024). In addition to that, AMPs can also exert additional effects such as immune modulation or disruption of biofilms, and binding to important cellular component of the microbes.

Traditional approaches in the discovery of antimicrobial peptides are powerful but have limitations. They require a known biological source and often overlook cryptic peptides encoded in genomes or microbiomes of uncultivated organisms (Santos-Júnior et al., 2024). They also tend to rediscover common AMP families and rarely explore truly novel sequence space. Rational design efforts such as modifications to improve stability, reduce toxicity, or target specific pathogens and synthetic libraries such as combinatorial peptide arrays have enhanced the repertoire, but the sequence space is astronomically large and empirical screening is slow.

**FIGURE 2:**
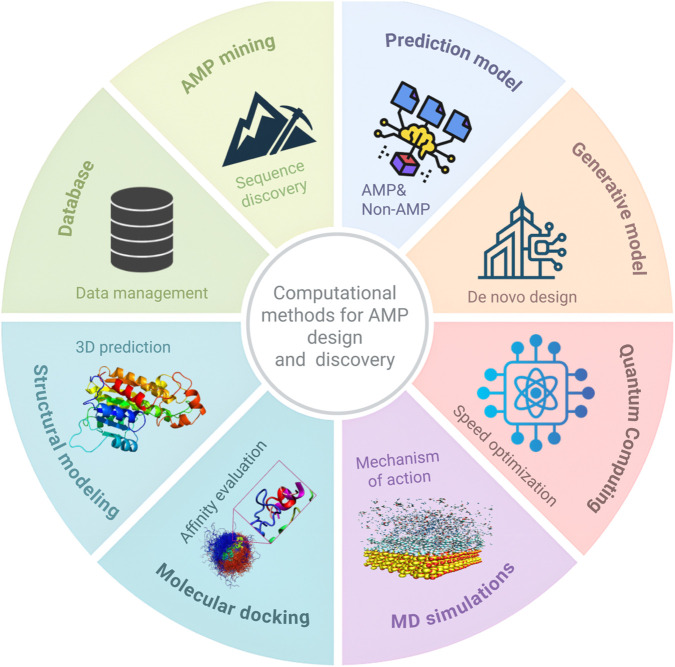
Computational methods in antimicrobial peptide (AMP) design. Database: Curated AMP sequences, structures, and activity. Mining: Algorithms identify potential AMPs. Prediction: Classify candidates. Generative models: Deep learning generates new peptides. Quantum computing: Accelerates discovery. MD simulations: Explore mechanisms of action. Molecular docking: Predicts binding modes and affinities. Structural modeling: 3D structure prediction. Created in BioRender. Allwell, E. (2025) https://BioRender.com/wawyedl.

Computational approaches are advancing in multiple directions (Figure 2). A computational modeling technique can characterize peptide target interactions and identify top promising peptides, while modern artificial intelligence approaches, such as large language models and graph neural networks, capture interrelations in sequence activity relationships spanning old and new methodologies. Quantum computing, for example, is emerging as a possible adjunct to speed up the discovery process (Li et al., 2024; Kumar et al., 2024; Blunt et al., 2022).

Despite this rapid technological evolution, most existing review articles on computational AMP discovery study individual strands, as a result, the target audience is left with a fragmented knowledge of the interplay between the approaches. This current review took the initiative to fill this gap by unifying logic of data-driven AMP modeling, large scale peptide archive mining, AI AMP design, multiscale modeling, and early-stage quantum algorithm fusion. It further elucidates the synergies among these computational approaches, critique their limitations, and outline how they can be combined to address current challenges in antimicrobial resistance (AMR).

## Big data approaches and databases in antimicrobial peptide discovery

The age of genomics and metagenomics has pioneered enormous “big data” catacombs from which AMPs can be mined. Everything from the environmental sequences of the soil and sea to gut microbiomes and genomic data from both pathogenic and commensal organisms provides trillions of DNA reads. Modern computation methods can scan these data for open reading frames that encode short cationic peptide sequences and rank them for their potential bioactivities. For example, small open reading frames (sORFs) from microbial genomes can be screened for AMP-like features such as charge and hydrophobicity before the peptide is synthesized and tested.

Public repositories are accelerating this process by making these datasets much more accessible. A good example is NCBI GenBank (https://www.ncbi.nlm.nih.gov/genbank/) which is one of the most used repositories, containing elaborate genomic and transcriptomic datasets from which candidate AMPs can be mined (Musin and Asyanova, 2025). Another example of these databases is the UniProt Knowledgebase (UniProtKB: https://www.uniprot.org/uniprotkb) which combines protein sequences from many different sources and curates them into a single, high-confidence, non-redundant collection database. It also provides functional annotations, activity data, and cross-links to genomic context of the sequences, serving as an indispensable database in sequence-based AMP discovery. Some other commonly use databases are Ensembl (Birney et al., 2004), GenBank (Benson et al., 1993), RefSeq (NCBI) (O’Leary et al., 2016), DDBJ (Tate et al., 2002), to mention a few.

Resources of metagenomic data can also be accessed through these public repositories. These sets of data specifically open the possibility to explore microbial communities in their uncultured states, thus extending the range of exploration far beyond the traditionally cultured genomes, allowing the discovery of new classes of antimicrobial peptides. A recent study used tens of thousands of public metagenomes to predict prokaryotic AMPs systematically (Santos-Júnior et al., 2024). Using deep learning, they trained a classifier on known AMPs and made use of the model to predict AMP sequence from their datasets. This process produced around 9.9 million candidate AMPs, which, after redundancy filtering, yielded a catalog of 863,498 unique candidate sequences. From this 100 AMPs were synthesized and tested against clinically relevant drug-resistant pathogens and human gut commensals both *in vitro* and *in vivo*, and a total of 79 active AMPs were discovered (Santos-Júnior et al., 2024).

In a similar spirit, the human gut microbiome had been mined for peptide antibiotics (Santos-Júnior et al., 2024). Seventy-eight peptides were synthesized and screened for antimicrobial activity *in vitro*, with 70.5% displaying antimicrobial activity. In a similar study researchers showcased the utility of combining NLP methods and large microbiome data for the mining for AMPs (Ma et al., 2022). Other examples of data driven discovery include targeted searches in specific environments. For instance, researchers have begun mining soil, ocean, and plant associated metagenomes for AMPs ^10^.

Aside from that, specialized AMP databases curate known antimicrobial peptides with detailed annotations. Databases like Antimicrobial Peptide Database (APD), CAMP, DRAMP, and others (Table 1) curate thousands of experimentally validated AMPs, with annotations of structure, activity spectrum, and modifications. These serve as training sets and benchmarks for ML models. Modern pipelines often start with these databases to train classifiers or generative models.

**TABLE 1:** Some publicly available Antimicrobial Peptide (AMP) Databases.

Database	Entries (approx.)	Features	Link
APD3 (Wang et al., 2016)	>5,000	Natural AMPs, synthetic, and predicted AMPs with the following function activities	https://aps.unmc.edu
CAMP R4 (Gawde et al., 2023)	>24,000	Sequences, 933 3D structures, 2,143 patents, activity assays (MIC/hemolysis), motifs	https://camp.bicnirrh.res.in
DBAASP v3 (Pirtskhalava et al., 2021)	>23,000	contains information on ribosomal, non-ribosomal, and synthetic peptides that show antimicrobial activity as Monomers, Multimers, and Multi-Peptides	https://dbaasp.org/home
dbAMP (Jhong et al., 2022)	>35,000	updated resource of antimicrobial activity and structural annotation of peptides in the post-pandemic era	https://awi.cuhk.edu.cn/dbAMP/
DRAMP 4.0 (Ma et al., 2025)	30,260	curated database harboring diverse annotations of AMPs including sequences, structures, activities, physicochemical, patent, clinical and reference information	http://dramp.cpu-bioinfor.org
AMPDB v1 (Mondal et al., 2023)	>10,715	comprehensive database of anti-microbial peptides, containing an almost exhaustive list of anti-microbial peptides known currently, and extensive curated information	https://bblserver.org.in/ampdb/

### Molecular docking in AMP discovery

A critical step in AMP discovery is the identification of promising peptide candidates with potential biological activity. These activities in the past have been carried out using high throughput screening (HTS) which involves exposing numerous peptide or peptide mimetic libraries to biological assays which are relevant to microbes. HTS, while providing concrete biological activity assessments, is constrained by several limitations. The screening of exhaustive libraries is a highly expensive and time intensive process, in addition to the limited chemical space peptide of the available libraries. To make matters worse, not every antimicrobial assays can be performed at scale. This results in a trade off in the density and relevance of the information collected.

In this context, molecular docking serves as a key computational approach and has become the dominant method in virtual screening (VS) for antimicrobial peptide (AMP) discovery. The docking process allows the assessment of peptide and its corresponding targets *in silico*. This method provides highly desirable information for peptides with higher interacting surfaces with a target. The biological targets from this method usually come from validated bacterial proteins, membrane spanning receptors, or enzymes which can be accessed via public databases such as protein databases. When experimental structures are unavailable, high-confidence models predicted by AlphaFold and other structure prediction tools can be used. Together, these features increase how useful, reliable, and informative the method is.

In the past decade many studies have used docking to discover or optimize AMPs. These include *de novo* design of peptide sequences, screening natural peptide libraries against new targets, and repurposing known AMPs. For example, 52 novel peptides targeting MRSA were designed by computational methods. The candidates were screened by physicochemical properties and then each peptide was docked against MRSA specific proteins (Sinoliya et al., 2025), after which molecular dynamic simulation was carried out to validate interactions before further testing. Similarly, another study combined machine learning and docking to find antischistosomal AMPs. AMPs were filtered by ML for suitable properties, then 18 candidates docked to the glyceraldehyde three phosphate dehydrogenase (GAPDH) and triose phosphate isomerase (TPI) protein interaction (PPI) in *Schistosoma mansoni* using HADDOCK (Isa and Kappo, 2025).

Other studies have docked natural AMPs against new targets to propose repurposing. For instance, a recent study demonstrated the docking of a panel of established AMPs to human receptors associated with infection-related cardiovascular disease (Dermawan and Alotaiq, 2025). The study was able to highlight the therapeutic potential of tachystatin, pleurocidin, and subtilisin A in targeting infection related pathways in cardiovascular diseases via molecular docking among other powerful bioinformatics tools (Dermawan and Alotaiq, 2025).

**FIGURE 3:**
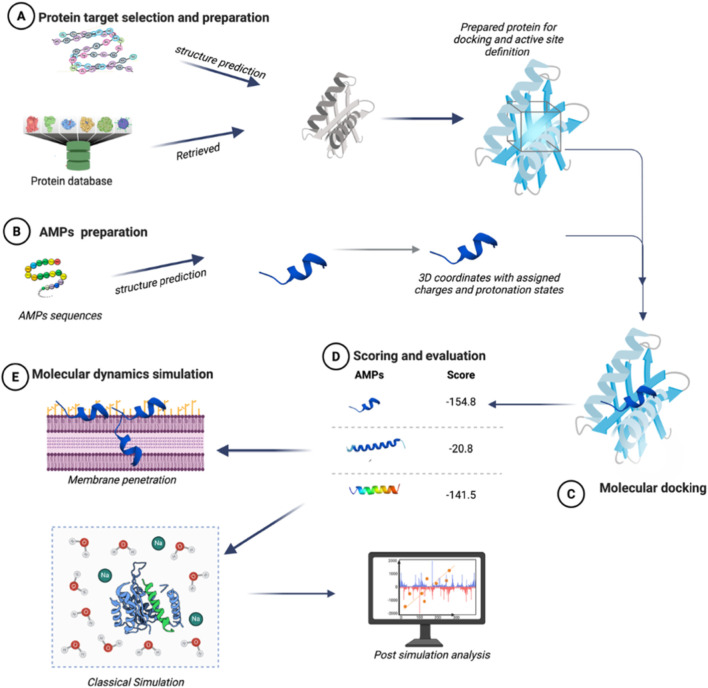
Molecular docking and dynamics in AMP discovery. **(A)** Protein retrieval or modeling and active site definition. **(B)** AMP sequence preparation and 3D structure generation. **(C)** Docking to predict peptide–protein binding orientations and interactions. **(D)** Ranking docked complexes by scoring functions. **(E)** Molecular dynamics simulations to study AMP mechanisms, including binding, membrane perturbation, pore formation, and interaction stability. Created in BioRender. Allwell, E. (2025) https://BioRender.com/mcd4znh.

Simple docking tools such as AutoDock and Vina, while fast and popular, do not give enough internal flexibility to the peptide chains, so they are prone to missing the correct binding pose of longer peptides, and favour linear docking which is a much less complex pose (Rentzsch and Renard, 2015). More advanced docking tools such as HADDOCK, Rosetta FlexPepDock (Table 2), and other deep learning and machine learning platforms manage to handle internal flexibility better, but at the cost of higher computational resources and, at times, requiring some sort of advanced user configuration. It is common practice for users to run docking tools in parallel or to do some form of molecular dynamics simulation (which is covered in the next subsection) to augment the confidence of the docking predictions (Figure 3).

**TABLE 2:** Molecular docking tools for antibacterial peptide discovery.

Tool	Link	Docking approach
HADDOCK (Dominguez et al., 2003)	https://www.bonvinlab.org/software/haddock2.4	Information-driven docking, supports ambiguous restraints and flexible refinement
HPEPDOCK (Zho et al., 2018)	http://huanglab.phys.hust.edu.cn/hpepdock/	Blind hierarchical peptide docking; global search with peptide flexibility
HDOCK (Yan et al., 2020)	http://hdock.phys.hust.edu.cn/	Protein-protein and protein-DNA/RNA docking based on a hybrid algorithm
ClusPro PeptiDock (Jones et al., 2022)	https://cluspro.org/peptide/index.php	FFT rigid-body peptide docking method, PIPER followed by clustering
MdockPeP (Xu et al., 2018)	http://zougrouptoolkit.missouri.edu/mdockpep	Global *ab initio*docking using multiple peptide conformers
pepATTRACT (de Vries et al., 2017)	https://bioserv.rpbs.univ-paris-diderot.fr/services/pepATTRACT/	Blind docking with coarse-grained search + atomistic refinement
CABS-dock (Kurcinski et al., 2015)	http://biocomp.chem.uw.edu.pl/CABSdock	Flexible peptide docking
Rosetta FlexPepDock (London et al., 2011)	http://flexpepdock.furmanlab.cs.huji.ac.il/	High-resolution refinement of peptide–protein complexes
PIPER-FlexPepDock (Alam et al., 2017)	http://piperfpd.furmanlab.cs.huji.ac.il	fragment-based protocol for high-resolution global peptide-protein docking
GalaxyPepDock (Lee et al., 2015)	http://galaxy.seoklab.org/pepdock	Template-based docking with interaction similarity and energy optimization
LightDock (Jiménez-García et al., 2018)	https://lightdock.org/	Swarm intelligence–based flexible docking; supports peptide and membrane docking
AutoDock CrankPep (ADCP) (Zha et al., 2019)	https://github.com/ccsb-scripps/ADCP	Sequence-to-structure peptide docking; samples conformations directly from peptide sequence

Also, a more recent approach which is the integration of machine learning with molecular ocking to develop a new approach of AMP prediction and discovery (see below). Recent reviews also shows that machine learning approaches offer more flexibility to the docking prediction, data driven frameworks to refine the predictions and docking to increase enrichment rate, thereby enhancing the prioritization of peptides (Khamis et al., 2015; Ballester et al., 2010; Crampon et al., 2022; Ahmed et al., 2022).

### Molecular simulations of AMPs

Alongside big data approaches, molecular docking (MD) and molecular simulations also facilitate mechanistic understanding. At the mechanistic level, molecular simulations give a crucial understanding of AMP action. They can illuminate function/structure relations and help refine or validate AI hypotheses. For many years, atomistic and coarse-grained simulations have been employed to investigate the interaction of AMPs with membranes or other molecular targets (Yoon et al., 2024; Graham et al., 2017; Do et al., 2018; Ulmschneider and Ulmschneider, 2018; Suter J et al., 2009; Shi et al., 2006).

Classical MD simulations give insights on AMPs and how they attach to membranes, create pores, or aggregate. MD simulations can also be used to analyze the synergistic peptide effects and compare the effects of different peptide variants. An example of MD in recent research was a MD study of hetero-oligomers of AMPs, in particular, where peptide aggregates were simulated in an aqueous solution and near a model membrane. Importantly, these simulations demonstrated different residue compositions resulted in different modes of membrane binding, providing an explanation on why some combinations synergistically disrupt membranes more than others (Yoon et al., 2024).

Another example of MD simulation is Coarse Grained (CG), such as the MARTINI force field, that allows simulation of larger systems and longer timescales. This is useful for studying complete pore formation or peptide distribution on membranes (Richardson and Van Lehn, 2024). This approach can be used to model the collective behavior of dozens of peptides on a lipid bilayer, showing phenomena like peptide-induced membrane thinning, curvature, or domain effects, as well as the discovery of the most potent AMP among the batch.

Steered Molecular Dynamics (SMD) can be used to investigate the ability of AMPs to penetrate membranes. SMD introduces an external bias to drive a molecule across the membrane. Steered Molecular Dynamics (SMD) can be used to investigate the ability of AMPs to penetrate membranes (Yu et al., 2026). SMD introduces an external bias to drive a molecule across the membrane. This approach helps to accelerates rare events and provides mechanistic insight into insertion pathways, making it valuable for comparative screening. However, results are sensitive to pulling speed, spring stiffness, and choice of pulling atom versus centre of mass (Do et al., 2018).

MD is not limited to membranes. Some AMPs work by binding intracellular targets such as enzymes or nucleic acids. MD simulations of AMPs bound to bacterial protein targets like signal peptidases or chaperones can also be carry out to reveal how peptide conformational flexibility and electrostatics contribute to specific binding (Do et al., 2018). Ultimately, MD complements the static models of AI by adding dynamics and thermodynamics, One of the still unsolved practical problems within this area is the speed of the calculations. MD simulations, for instance, are, as of today, still orders of magnitude slower than molecular docking due to their high computational demands that prohibit routine simulations greater than a microsecond in length (Durrant and McCammon, 2011), this is why MD is still largely confined to besting the most promising candidates or studying the mechanisms rather than brute force screening (Durrant and McCammon, 2011). However, advances such as GPU accelerated MD, quantum computing, and machine learned force fields are expanding what is tractable.

### Quantum computing in drug design and AMP optimization

Quantum computing introduces a transformative paradigm in computation by employing qubits instead of classical bits. Qubits are different from classical bits, which can only hold either a zero or a one, since they can exist in a superposition state in which they can hold multiple values at once (Nofer et al., 2023). This ability to encode multiple values in a single qubit along with the phenomena of entanglement and quantum parallelism allows the computation of multiple values simultaneously. Which could allow them to find solutions to problems which classical computers are unable to solve efficiently, which may eventually accelerate aspects of the drug discovery process.

Recently, there has been several emerging quantum approaches in drug discovery include quantum machine learning models that can encode high-dimensional molecular and protein features into quantum states, leveraging superposition and entanglement to capture complex interactions more effectively than classical embeddings (Pallavi et al., 2025). One recent study introduced a model that integrates a quantum support-vector-regression with a classical kernel, enabling accurate prediction of binding affinities on standard datasets. The authors note that quantum feature maps may generalize better with limited data, hinting at improved drug-target screening performance compared to purely classical methods.

Quantum computers are being applied to optimized molecular simulation. For instance, a hybrid pipeline has been built where VQE on a two-qubit active space can capture intricate bonding and reaction profiles, which could offer more detailed insights than classical approximations in limited or simplified systems (Li et al., 2024). Quantum methods are also being explored for molecular docking especially in ligand conformation searches and scoring. A recent hybrid *QCBM–LSTM* generative model was benchmarked on docking tasks: it generated many high-quality ligand candidates and demonstrated performance in docking tasks that, in some benchmark cases, was comparable to classical generative models (Gha et al., 2025). In addition, quantum computing is being explored for potential applications in the hit-to-lead stage, to improve property prediction and generating novel compounds. One striking example is a hybrid quantum classical generative model was used to design KRAS inhibitors (Kwon and Kim, 2025). Separately, quantum ML has been applied to lead property optimization, in a recent study, a quantum kernel classifier achieved high accuracy in predicting absorption, distribution, metabolism, and excretion (ADME) (Bhatia et al., 2023).

### Artificial intelligence in AMP design

**FIGURE 4:**
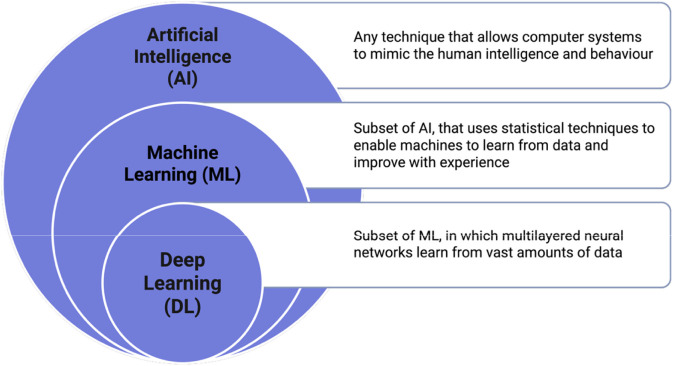
Artificial Intelligence overview. Created in BioRender. Allwell, E. (2025) https://BioRender.com/fxhmofs.

**FIGURE 5:**
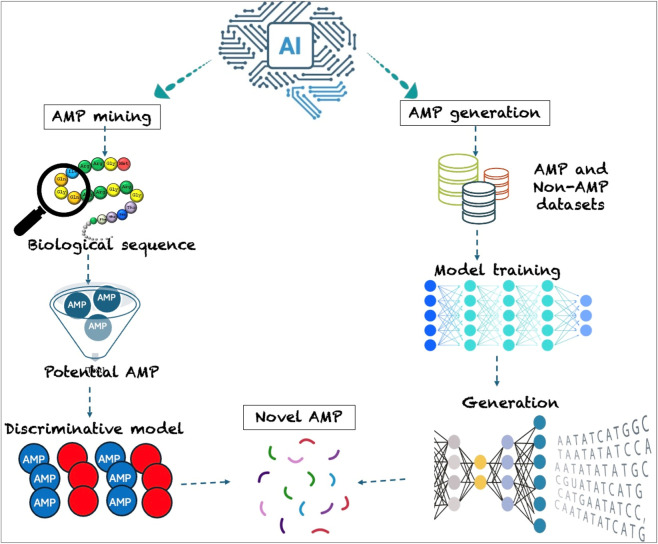
Artificial intelligence approaches in antimicrobial peptide Discovery. Created in BioRender. Allwell, E. (2025) https://BioRender.com/5ie01vo.

In recent time, use of Artificial intelligence (AI) which broadly encompasses machine learning (ML) and deep learning (DL) is becoming more popular, particularly in the area of designing and discovering AMPs (Figure 4). Machine learning approaches such as support vector machines, decision trees, and random forests have shown great potential in learning patterns. With DL, more sophisticated neural networks such as convolution/recurrent networks or transformers are utilized to learn complex sequence features unsupervised. In addition to that, AI in AMP research helps lessen the burden of research in the sequence of peptides by rapidly sifting through millions of candidate peptides to discriminate potential AMP from non-AMP (Figure 5).

### Predictive and discriminative models

Early antimicrobial-peptide (AMP) classifiers relied on hand-crafted features, such as amino-acid composition, physicochemical indices, and pseudo amino-acid compositions, combined with classical machine-learning algorithms. Support Vector Machines (SVMs) and Random Forests (RFs) were widely used to separate AMPs from non-AMPs. For examples a recent *in silico* approach used compositional residue features and tested 15 different machine-learning algorithms, including CatBoost, gradient boosting, extra trees, XGBoost, LightGBM, and RF, to classify AMPs and non-AMPs (Singh et al., 2021). In that study, the Boruta feature-selection method was employed to identify discriminative biological features, which were then used to construct the prediction model. Model performance was assessed through stratified tenfold cross-validation and further validated using an independent holdout test dataset (Singh et al., 2021). Further, tools like amPEPpy (2021) use RFs on global protein descriptors to classify AMPs in a more portable manner. Other classifiers like k-Nearest Neighbors or Naïve Bayes classifiers have also been used, but SVM and RF seem to be the most widely adopted. These former discriminative classifiers are good for scanning large libraries of peptides to find candidates but unable to create original sequences.

There has been a decisive shift toward deep neural networks that learn sequence features directly instead of relying on handcrafted descriptors. There has also been a focus on Convolutional Neural Networks (CNNs) and Recurrent Neural Networks (RNNs), especially LSTM and bi-LSTM architectures, with raw peptide sequences. For instance, iAMP-AttenPred (2023) uses pretrained BERT embeddings with a 1D-CNN, bi-LSTM, and attention layers, and shows state-of-the-art performance in classifying AMPs versus non-AMPs. Studies find that even relatively simple 1D-CNNs can match or outperform classical methods like SVMs and RFs when large training sets are available (Cai et al., 2025). Deep models are increasingly used for multi-task outputs, enabling simultaneous classification and potency prediction, and can be fine-tuned for regression to estimate antimicrobial activity levels.

Transfer learning via protein language models (PLMs) has emerged as a powerful strategy. Large pretrained models (ESM, ProtBert, TAPE, etc.) are trained on millions of protein sequences and capture amino-acid sequences; their embeddings can then be fine-tuned for AMP classification or MIC regression. For instance, BERT AmPEP60, a BERT-based model fine-tuned to predict minimum inhibitory concentrations (MICs) of AMPs against *Escherichia coli* and *Staphylococcus aureus* (Cai et al., 2025), leveraged self-attention to encode long-range sequence patterns. Embeddings from ProtT5 and similar PLMs improve AMP/non-AMP classification accuracy compared with traditional descriptors (Marquet et al., 2022).

Graph neural networks (GNNs) are another emerging approach. Peptides can be represented as graphs, with residues as nodes and edges connecting spatially adjacent residues, allowing incorporation of predicted or known 3D structural information. SGAC is a GNN framework tailored for imbalanced, structure-aware AMP classification (Wang et al., 2026). These models which consider structure can do more than simply anti-microbials prediction, they can also hypothesize the disruption of membranes and the targeting of membranes and cells (Fernandes et al., 2023). By merging structure types (helical, coil and mixed) and assigning a different model for each, the functional prediction can be significantly improved (Aguilera-Puga and Plisson, 2024).

Despite these advances, predictive models face important limitations. Data quality is a central challenge. Many reported AMPs lack rigorous MIC measurements, and negative examples (i.e., non-AMP peptides) are frequently assumed rather than experimentally confirmed. These issues introduce biases and can lead to over optimistic performance estimates on training data. To mitigate this, hybrid approaches combine sequence-based AI with human-curated rules or additional filters, for example, toxicity filters. Rigorous cross-validation on held-out test sets and benchmarking against independent datasets remain essential for robust model assessment.

### AMP mining strategies

Another power approach for discovery of AMPs is mining. The rapid growth of biological sequence data has accelerated AMP discovery through large-scale mining approaches. These strategies apply computational methods to genomes, proteomes, and metagenomes to predict novel AMPs. While early discoveries originated from amphibian skin secretions (Li et al., 2022), today’s vast sequence repositories provide far broader opportunities. Mining requires careful handling to minimize false positives and ensure predictions are experimentally validated. Most efforts focus on antimicrobial activity, though toxicity prediction is less frequently integrated due to its limited reliability.

Public databases now host millions of genomes and proteomes, offering rich resources for AMP discovery. For instance, the Global Microbial Gene Catalogue aggregates billions of open reading frames (ORFs) from thousands of metagenomes, clustered into hundreds of millions of species-level groups, and includes thousands of antimicrobial resistance (AMR) genes identified through homology searches.

Recent advances have expanded mining efforts beyond classical sources. The human proteome and microbiome have emerged as particularly promising reservoirs, given their clinical relevance and the relative safety of host-associated peptides (Wang, 2014). Deep learning approaches such as LSTM, attention mechanisms, and BERT have successfully mined the human gut microbiome, yielding numerous novel peptides with potent antimicrobial activity and less than 40% sequence similarity to known AMPs in recent years. Several of these candidates has also been validated experimentally and showed strong efficacy against antibiotic-resistant pathogens (Ma et al., 2022).

Alternative mining strategies have also focused on bacteriophage derived antimicrobials. A pipeline to extract AMPs from phage peptidoglycan hydrolases (PGHs) associated with ESKAPE pathogens has been developed. Using CNN and LSTM models, they generated ESKtides, a database containing over 12 million peptides with predicted antibacterial activity (Wu et al., 2024).

Bioinformatic tools can scan bacterial genomes for gene signatures to mine AMPs that are ribosomal synthesized and the one in which their post-translationally modified peptides encoded within biosynthetic gene cluster. For example, 50,000 bacterial genomes have been mined for lanthipeptides (lanthionine-containing) and a novel peptide that was potent against multidrug-resistant *S. aureus*, including MRSA and linezolid-resistant strains, was discovered (Cheng et al., 2024). Other approaches in this domain also harvest DNA from entire microbial communities (metagenomics) (Pérez-Cobas et al., 2020) and predict AMPs from fragmented sequence data. Other metagenomic mining studies have targeted soils, ocean samples, and even industrial wastes. Upon analyzing sewage sludge metagenomes by ML, over 16 million candidate AMPs have been predicted (Xu et al., 2024).

### Generative AI strategy

Beyond prediction, AI is being used to design new AMPs from scratch. Generative models can sample novel sequences optimized for desired properties. Early efforts used genetic algorithms or basic generative methods, but modern AI has greatly expanded this capability. Two broad classes of generative models are in use: generative adversarial networks (GANs) and variational autoencoders (VAEs) or related variational/diffusion methods.

GAN based frameworks have shown promise in AMP design. For instance, the Feedback GAN (FBGAN) model was introduced to iteratively generate DNA sequences encoding AMPs (Ying et al., 2025). In FBGAN, a generator network creates candidate peptide encoding sequences, and a discriminator (or a classifier) scores their AMP likeness; high scoring sequences are fed back to further train the generator. This allows the network to explore sequence space while being guided by predicted AMP functionality. More recently, improvements have been made on this concept by integrating a regression model for MIC and multi label classification for multiple antimicrobial activities into the GAN framework (Ying et al., 2025). The hybrid model produced peptides predicted to be not only antibacterial but also antifungal, antiviral, etc. Experimental validation showed that their system efficiently generated peptides with strong predicted activity in multiple categories (Ying et al., 2025).

VAEs and diffusion models represent another approach. VAEs compress peptides into a continuous latent space; by sampling and decoding points in this space, new sequences can be generated. A striking example is the work noted above, where a binary VAE was coupled with quantum annealing in latent space (Dean and Walper, 2020). Other researchers have used VAEs without quantum parts: for instance, AMP Generator, a VAE trained on known AMPs, can interpolate to create intermediate sequences between known families (Dean and Walper, 2020). Diffusion models (which gradually add noise to sequences and then learn to reverse this process) are also emerging. AMP Diffusion, combining a latent diffusion model with a protein language model, has been used to generate structurally diverse AMPs (Cheng et al., 2024). This approach decouples sequence generation from property control, enabling the user to modulate peptide length and novelty.

Large language models (LLMs), pre trained on huge protein databases, are increasingly applied to peptides. Just as Generative Pre-trained Transformer 3 (GPT-3)revolutionized text generation, models such as ProtGPT2 or ProteinBERT trained on millions of protein sequences can be fine-tuned to generate peptide sequences. A handful of studies have begun to use such foundation models in AMP design. For example, an AMP specific LLM (AMP GPT) has been used as a foundation model called AMP Designer (Wang et al., 2025). In only 48 days, AMP Designer produced 18 *de novo* peptides that were synthesized and tested; 17 of 18 were active (94.4% hit rate). The two top candidates were effective in mouse infection models with low toxicity, illustrating that LLM based design can rapidly yield potent AMP lead (Wang et al., 2025). This is one of the fastest and most efficient discovery pipelines reported: 11 days to generate candidates and 48 days end to end, markedly quicker than typical drug discovery timelines. Another recent example used a foundation model approach (protein LLM embeddings + classifier) to screen billions of peptides and identified broad spectrum AMP candidates (Wang et al., 2025).

Together with predictive, mining, generative AI is also shifting AMP discovery from screening to synthesis. The success of these models hinges on accurate predictive scorers, most generative pipelines incorporate an AI predictor for AMP activity (or hemolytic toxicity) to filter or steer generation. Optimization algorithms such as Bayesian, Adam, gradient based in latent space, reinforcement learning are often added to fine tune sequences. The AI has also be use in recent years to address the open question on how to incorporate structure in AI design. Pure sequence-based models may miss nuances of conformation or stability. Some hybrid methods are structure aware, for instance, separate generative models for peptides have been built that were constrained to helical vs. coiled structures (Aguilera-Puga and Plisson, 2024). Others have proposed using AlphaFold or Rosetta to predict 3D structures of AI generated peptides and reject those unlikely to fold or bind membrane (Fang et al., 2025). Further integration of AI with biophysical modeling is likely to be forthcoming. For example, generative models could be trained to produce sequences that are predicted molecular interactions to bind a target membrane or protein, this will be a paradigm shift in personalized therapy.

### Bottlenecks and challenges

The role of high-quality datasets is paramount in AI driven antibiotic discovery, and most computational models depend on known AMP databases for training. These databases are incomplete and biased toward well studied peptides, making it hard to learn the full diversity of mechanisms. Negative data (non-AMP peptides) are especially scarce, often assumed rather than experimentally confirmed. This can lead to inflated model performance and many false positives.

Another challenge in computational AMP discovery is that antimicrobial activity is not the only goal: toxicity to host cells (e.g., hemolysis), stability (protease resistance), solubility, serum interactions and manufacturability also matter. This makes multi-objective optimization complex because improving one property often worsens another.

Another issue is resource challenges for training models or keeping datasets. While sequencing data and compute resources are abundant, large scale ML training (LLMs) and quantum algorithms require specialized hardware. Training a large peptide model or running MD on many candidates is expensive. Access to HPC clusters and cloud GPUs is not universal, potentially limiting smaller laboratories, and those from lower resource countries. Similarly, quantum devices are still scarce. Methodological efficiencies (e.g., transfer learning) and shared cloud resources help, but resource constraints are real.

AI models trained on existing data may not generalize well to novel sequences far from training examples. There is a risk of overfitting to known AMP motifs. Some recent papers address this by explicitly testing models on “out of distribution” sequences and by engineering novelty promoting regularization (Porto et al., 2017; Das et al., 2021; Madani et al., 2023). However, there is a lack of systematic understanding of the true sequence diversity of AMPs in nature, so it’s hard to guarantee that models will find genuinely new scaffolds.

In summary, while computational methods have lowered some barriers, others have emerged. Improving data (both quantity and quality), enhancing model explainability, and integrating multi objective constraints are ongoing efforts. Likewise, as quantum methods develop, understanding when they truly outperform classical algorithms is a current research topic. Researchers continually stress that AI/ML and simulations are tools to augment, not replace, experimental science (Wang et al., 2025). Overcoming these bottlenecks will require continued interdisciplinary collaboration among computer scientists, biochemists, and clinicians.

### Synergistic framework for unified computational AMP discovery

Instead of considering big-data mining, predictive modelling, AI-driven sequence design, multiscale simulations, and quantum algorithms as separate tools, we believe that these techniques are at their best when they are carefully combined into a synergistic, end-to-end discovery pipeline. In this seamlessly integrated system, machine learning models use curated sequence-activity relationships to identify the most promising candidates, while extensive mining helps to increase the chemical diversity explored.

Gen AI components then delve further into and enlarge this representation space by generating new AMP-like sequences that fit multi-objective user-defined criteria, such as effectiveness against priority pathogens, low hemolysis, and good physicochemical stability. Multiscale biophysical methods, from structure prediction, molecular docking, enhanced-sampling MD, to coarse-grained membrane simulations, offer a mechanistic filter that can validate the predicted activity, elucidate probable modes of action, and find candidates with good selectivity and robustness under physiologically relevant conditions.

Lastly, quantum algorithms at the early phase and quantum-inspired optimization methods provide a few specialized solutions to very tough subproblems, e.g., combinatorial sequence optimization in huge design spaces or the exploration of complex energy landscapes, and they may be integrated as optional modules within the overall generative and scoring framework when the appropriate hardware or emulation platforms are accessible.

### Future prospects and outlook

Going forward, we anticipate computational AMP discovery to be dominated by integrated, closed-loop pipelines which will connect big-data mining, predictive modelling, AI-guided sequence design, and multiscale biophysical validation in one complete cycle. It is recognized that the greatest advances will probably come through the convergence of these strands in practice, such as laboratory-integrated. Achieving this goal will necessitate consistent commitment to data standards that allow Findable, Accessible, Interoperable, Reusable (FAIR) repositories, open and reproducible computational tools, and fair access to computing resources, especially for labs in low--and middle-income countries that are the most affected by antimicrobial resistance. If these scientific, infrastructural, and policy challenges are solvable, then the unified computational paradigm described in this paper could be a steppingstone to the much-awaited “golden era” of antibiotic discovery based on rational, data-driven AMP development.

## Conclusion

Recent technological developments such as big data, artificial intelligence and quantum computing are transforming the field of discovery related to AMPs from a largely off-hand and somewhat serendipitous process into a fine-tuned discipline with real precision. Now, precisely tailored AI platforms can search thousands datasest for potential antimicrobial peptides in a relatively short time. Making it possible to truncate discovery periods from decades to months. In several AI guided studies, success is noted to be as high as 94%. While the potential of these technological advancements is exciting, there exist several challenges. The inherent quality and bias of the training databases continues to limit the potential for model generalization. The complex multi-objective optimization problem of balancing antimicrobial potency with toxicity, stability, and manufacturability remains a barrier.

The ultimate goal of this computational revolution is not to be assessed by the sophistication of the algorithms, or the number of data points processed, but rather, the actual number of lives saved, and ultimately, the antimicrobial susceptibility reestablished within clinical practice. Those outcomes collectively speak to why there is a need to carry on this research. With the emerging combination of computational and experimental drug discovery, new and real opportunities to together push back against ever-evolving pathogens, and to sustainably control antimicrobial resistance which remains perceived as an unavoidable facet of modern medicine have been created.
